# Family Satisfaction of Polytrauma Patients in Intensive Care Unit at a Tertiary Care Center

**DOI:** 10.7759/cureus.65702

**Published:** 2024-07-29

**Authors:** Vipin K Singh, Azin Ahmad, Vaibhav Jaiswal

**Affiliations:** 1 Anesthesiology, King George's Medical University, Lucknow, IND; 2 Trauma and Acute Care Surgery, King George's Medical University, Lucknow, IND

**Keywords:** patient care, quality of life (qol), decision making, intensive care unit, family satisfaction, polytrauma patient

## Abstract

Background: Family members play a crucial role in ICU patients' treatment and decision-making, despite the stress and uncertainty they may experience, ensuring high-quality medical care. Providing comfortable spaces with noise-reducing techniques can boost family satisfaction. Further research is needed to support families in intensive care units (ICU). This study aims to evaluate family satisfaction and decision-making in polytrauma patients in the ICU, identify improvement opportunities, and analyze demographic and socioeconomic factors influencing satisfaction.

Methods: This cross-sectional study was conducted at King George's Medical University, Lucknow, over a period of one year. A total of 66 patients, aged between 20 and 70, their family members, and those who gave written informed consent were included. Exclusion criteria included those who died within 48 hours of ICU admission or did not give consent. Patient characteristics, such as age, sex, Acute Physiology and Chronic Health Evaluation (APACHE) II score, and hospital stay length, were also collected. The family satisfaction in the intensive care unit (FS-ICU) questionnaire, consisting of 24 items with five Likert response options, was used to assess satisfaction levels in ICU care and decision-making.

Results: A study of 66 patients which included 78.79% male and 21.21% female. The majority of the patients (66.67%) lived with their family members. The mean ICU stay was 13.03 days, with an APACHE score of 17.39. The results showed that families were very satisfied with a considerable portion of the ICU stay. The overall satisfaction score was 57.00. Families were less satisfied with the atmosphere in the ICU and involvement in the decision-making process. The satisfaction scores were comparable for both genders, except for the time taken to respond to questions, which was significantly higher for women.

Conclusion: Although families were very satisfied with the ICU stay, several areas were identified as having potential for improvement. The present study shows that the quality of treatment and communication during hospitalization is a major factor in the need for follow-up care. This underlines the need for a constant focus on communication skills in the training of nurses and doctors and in their practical training in the ICU. Participation in decision-making, especially by family members of survivors, was identified as an area for improvement. We recommend more research to be conducted in India focusing on family satisfaction with involvement in the decision-making in ICU considering the unique racial, cultural, ethnic, and linguistic differences in India.

## Introduction

Today, patient experience, opinion, and satisfaction are regarded as decisive quality factors for high-quality medical care. This is true for most patients who are admitted, but for those who are admitted to the ICU, the situation is quite different. Since most ICU patients are extremely ill, they cannot fully appreciate the care process and cannot actively participate in decisions about treatments and medical procedures. In such cases, family members and loved ones are crucial in their decision-making process. They cannot fully appreciate the care process and actively participate in decisions. Family members often experience the ICU experience as stressful, uncertain, and frightening but wish to be involved in the patient's treatment and decision-making [1].

Family engagement is crucial for holistic care for critically ill patients, and ICU nurses must meet their families' needs. The latest technology is used to treat and care for these patients, who may experience real events, vague memories, and hallucinations due to ICU delirium [2-4]. Family members play a crucial role in ICU patient care, acting as mediators and health-promoting resources. However, research indicates that family members may experience sadness, post-traumatic stress disorder (PTSD), or post-intensive care syndrome (PICS) [5-7].

Families in the ICU face challenges due to uncertainty about patient conditions, treatment, and prognosis [8]. They want to participate in care but need improvement in receiving support. Nurses' support is crucial for understanding and coping, but patients may struggle to evaluate their stay due to illness and treatment. Research into family satisfaction has increased in recent years [9-11].

In India, medical resources are inadequate for family-centered care, prompting the need for surveys on family satisfaction scores (FSs) to measure patient care and their involvement in decisions about ICU patients [12].

To ensure that the treatment meets both the patient's and the family's needs, the family's satisfaction with the patient's care during the ICU stay can be a crucial piece of information that contributes to the overall quality improvement of the ICU [13]. There is not much published quantitative research on family satisfaction. More information is important to assess the transferability of results between national and international research and to protect the needs of family members. It is crucial to examine the ways in which different demographic factors influence family members' satisfaction scores [14-16].

The study aimed to assess family satisfaction and decision-making in polytrauma patients in ICU using a questionnaire - family satisfaction in the intensive care unit questionnaire (FS-ICU-24), identify improvement opportunities, and assess demographics and socioeconomic factors influencing satisfaction and decision-making.

## Materials and methods

The study was registered with the Clinical Trials Registry of India (CTRI/2023/06/054525) and conducted in the Department of Anesthesiology at King George's Medical University, Lucknow. The prospective randomized observational study lasted for one year, involving patients aged 20-70, all family members of patients admitted to the ICU for more than or equal to 48 hours, and those who gave written informed consent. Exclusion criteria included those who died within 48 hours of ICU admission or did not give consent.

The patient's next of kin (NOK) was designated as the pivotal contact person, and details were documented on the nursing chart at admission. The FS-ICU questionnaire was given to the NOK after 48 hours of ICU admission, and they were asked to complete it before the patient's ICU discharge. Family members were asked to provide data on their age, sex, relationship to the patient, frequency of visits, prior knowledge about the patient's health status, and ease of travel from home to the ICU. Patient characteristics, such as age, sex, Acute Physiology and Chronic Health Evaluation (APACHE) II score, and hospital stay length, were also collected.

A short version of the FS-ICU questionnaire consisting of 24 items was used. Each of these items contained five Likert response options ranging from “poor” to “excellent.” This survey estimated two broad sections. The first section mainly assessed satisfaction levels over a wide range of domains related to ICU care. The second section concentrated more on satisfaction with decision-making. FS-ICU was available in English and Hindi (the FS-ICU 24 was translated into Hindi and back-translated to English in line with the World Health Organization’s guidelines but has not yet undergone validity and reliability testing in India). For study participants who were not comfortable with English/Hindi, FS-ICU was translated verbally by a ward nurse who did not render services in the ICU. A nurse not working in the ICU was chosen as we did not want the nurse to influence the response to the questionnaire. The study aimed to understand the factors influencing patient satisfaction and improve healthcare delivery.

Individual responses of the FS-ICU were transformed to a scale between 0 and 100. Higher scores on the scale indicated greater satisfaction. The FS-ICU provides the following satisfaction scores: FS-ICU/care (satisfaction with care), FS-ICU/DM (satisfaction with information/decision-making), and FS-ICU/total (overall satisfaction with the ICU).

Sample size

Sample size was calculated on the basis of maximum variation in overall satisfaction on the Likert scale using the formula: n=k [(Z_^α ^_+ Z_^β^_ )^2^ (σ2 )]/d^2^, where σ=14.7, the maximum SD of overall satisfaction on the Likert scale; d=10% of mean satisfaction on Likert scale (64.94), the difference considered to be clinically significant; design effect k=1; type I error α=5% corresponding to 95% CI; type II error β=10% for detecting results with 90% power of study; n=66.

Statistical analysis

Demographic and clinical data of the patients, respondents, and answers to the questionnaires are presented as either mean (+/−SD) or frequency (percentage). After obtaining the responses (Likert scale) for each item of 66 respondents, it was linearly transformed into scores by using the formula: transformed value=((actual item value-lowest possible item value)/possible item range)×100. Using linear regression models, factors that are significantly associated with FS were obtained. Significant factors were represented as a coefficient (standard error) and P-values. A P-value of 0.05 was taken as the level for statistical significance.

## Results

Out of 66 patients, 78.79% were male and 21.21% were female. The percentage of 20-30 years, 31-40 years, 41-50 years, 51-60 years, and >60 years age group were 43.94%, 16.67%, 24.24%, 9.09%, and 6.06%, respectively. 80.30% had previous ICU admission. Of the 66 patients, 16.67% lived in the same city as the hospital. The frequency of living with the patient was 66.67% (Table 1).

**Table 1:** Distribution of patients according to demographic profile, ICU admission, and survival status ICU, intensive care unit

N=66	Parameters	N	%
Gender	Male	52	78.79%
	Female	14	21.21%
Age (years)	20-30 years	29	43.94%
	31-40 years	11	16.67%
	41-50 years	16	24.24%
	51-60 years	6	9.09%
	>60 years	4	6.06%
	Mean±SD	36.03±14.20	
Lives in the same city as the hospital	Yes	11	16.67
	No	55	83.33
Contact with patient	Lives with patient	44	66.67
	Weekly	3	4.55
	Monthly	11	16.67
	Less than one year	2	3.03
	Yearly	6	9.09
Previous h/o ICU admission	Yes	53	80.30
	No	13	19.70
Survival status	Yes	40	61.54
	No	26	38.46

The mean length of ICU stay was 13.03±6.03 days with a range of 3-29 days. The mean APACHE II score was 17.39±7.28 with a range of 4-32 days (Table 2).

**Table 2:** Details of length of ICU stay and APACHE II score ICU, intensive care unit; APACHE, Acute Physiology and Chronic Health Evaluation

	Mean	Median	SD	Minimum	Maximum
Length of ICU stay	13.03	12.00	6.03	3.00	29.00
APACHE II score	17.39	16.00	7.28	4.00	32.00

The survey showed high satisfaction scores for patient care, with 95.38% of participants rating the critical care staff's treatment of a family member's shortness of breath as good or better. The nursing staff's care was also praised, with a high coordination of care. The ICU atmosphere score was low. The medical care received was high. Participants felt involved, supported, and had control over their family member's care (Table 3).

**Table 3:** Descriptive data of the items of the FS-ICU-24 FS-ICU, family satisfaction in the intensive care unit

		n	%	Mean	±SD
1. How well did the ICU staff treat your family member with courtesy, respect, and compassion?	Poor	0	0.00	70.00	19.86
	Fair	3	4.55		
	Good	19	28.79		
	Very good	32	48.48		
	Excellent	12	18.18		
2. How well did the ICU staff assess and treat your family member's pain?	Poor	0	0.00	71.15	19.88
	Fair	3	4.55		
	Good	17	25.76		
	Very good	33	50.00		
	Excellent	13	19.70		
3. How well did the ICU staff assess and treat your family member's breathlessness?	Poor	0	0.00	67.31	18.18
	Fair	2	3.03		
	Good	25	37.88		
	Very good	31	46.97		
	Excellent	8	12.12		
4. How well did the ICU staff assess and treat your family member's agitation?	Poor	5	7.58	42.31	21.62
	Fair	22	33.33		
	Good	27	40.91		
	Very good	12	18.18		
	Excellent	0	0.00		
5. How well did the ICU staff show an interest in your needs?	Poor	7	10.61	43.46	24.72
	Fair	19	28.79		
	Good	26	39.39		
	Very good	12	18.18		
	Excellent	2	3.03		
6. How well did the ICU staff provide emotional support?	Poor	5	7.58	42.31	21.62
	Fair	22	33.33		
	Good	29	43.94		
	Very good	9	13.64		
	Excellent	1	1.52		
7. How well did the ICU staff co-ordinate the care of your family member?	Poor	0	0.00	73.46	18.16
	Fair	0	0.00		
	Good	19	28.79		
	Very good	32	48.48		
	Excellent	15	22.73		
8. How well did the ICU staff treat you with courtesy, respect, and compassion?	Poor	1	1.52	66.92	20.30
	Fair	3	4.55		
	Good	20	30.30		
	Very good	34	51.52		
	Excellent	8	12.12		
9. How well do you think the nurses cared for your family member?	Poor	0	0.00	73.08	17.85
	Fair	0	0.00		
	Good	19	28.79		
	Very good	33	50.00		
	Excellent	14	21.21		
10. How often did the nurses communicate to you about the family member's condition?	Poor	4	6.06	50.00	23.80
	Fair	15	22.73		
	Good	26	39.39		
	Very good	19	28.79		
	Excellent	2	3.03		
11. How well do you think the doctors cared for your family member?	Poor	0	0.00	79.62	17.62
	Fair	0	0.00		
	Good	11	16.67		
	Very good	32	48.48		
	Excellent	23	34.85		
12. The atmosphere in the ICU was?	Poor	8	12.12	36.15	21.66
	Fair	28	42.42		
	Good	22	33.33		
	Very good	8	12.12		
	Excellent	0	0.00		
13. The atmosphere in the waiting room was?	Poor	33	50.00	23.46	19.20
	Fair	20	30.30		
	Good	11	16.67		
	Very good	2	3.03		
	Excellent	0	0.00		
14. How satisfied were you with the level or amount of health care your family member received in the ICU?	Mostly satisfied	8	12.12	78.85	15.46
	Very satisfied	40	60.61		
	Complete satisfied	18	27.27		
15. How often did the doctors talk to you about your family member’s condition?	Poor	4	6.06	54.23	23.20
	Fair	8	12.12		
	Good	30	45.45		
	Very good	21	31.82		
	Excellent	3	4.55		
16. How willing were staff to answer your questions?	Poor	7	10.61	41.54	25.87
	Fair	26	39.39		
	Good	17	25.76		
	Very good	14	21.21		
	Excellent	2	3.03		
17. How capable were the intensive care staff of giving you explanations that you understood?	Poor	0	0.00	65.38	19.11
	Fair	3	4.55		
	Good	27	40.91		
	Very good	28	42.42		
	Excellent	8	12.12		
18. How honest do you think the information you received about your family member’s condition was?	Poor	1	1.52	65.38	20.10
	Fair	2	3.03		
	Good	27	40.91		
	Very good	30	45.45		
	Excellent	6	9.09		
19. How well did ICU staff inform about what was happening to your family member and why things were done?	Poor	0	0.00	68.85	21.66
	Fair	4	6.06		
	Good	22	33.33		
	Very good	26	39.39		
	Excellent	14	21.21		
20. How consistent was the information you received about your family member’s condition?	Poor	5	7.58	51.54	27.56
	Fair	16	24.24		
	Good	21	31.82		
	Very good	18	27.27		
	Excellent	6	9.09		
21. Did you feel included in the decision-making process?	Poor	33	50.00	30.77	33.88
	Fair	20	30.30		
	Good	11	16.67		
	Very good	1	1.52		
	Excellent	1	1.52		
22. Did you feel supported during the decision-making process?	Poor	12	18.18	41.92	30.97
	Fair	25	37.88		
	Good	14	21.21		
	Very good	9	13.64		
	Excellent	6	9.09		
23. Did you feel that you had control over the care of your family member?	Poor	20	30.30	40.77	32.94
	Fair	27	40.91		
	Good	11	16.67		
	Very Good	6	9.09		
	Excellent	2	3.03		
24. Did you have adequate time to concerns addressed and questions answered	1	39	59.09	89.62	12.42
	2	27	40.91		
	Total			57.00	4.34

Table 4 shows the comparison between men and women. The average satisfaction scores for all questions were comparable for men and women, with the exception of the reasonable time taken to respond to questions and concerns, which was significantly higher for women.

**Table 4:** Comparison of FS-ICU-24 between male and female FS-ICU, family satisfaction in the intensive care unit

	Male		Female		T	p-Value
	Mean	±SD	Mean	±SD		
1. How well did the ICU staff treat your family member with courtesy, respect, and compassion?	70.00	17.50	70.00	27.06	0.00	1.000
2. How well did the ICU staff assess and treat your family member's pain?	72.50	20.36	66.67	18.09	1.00	0.323
3. How well did the ICU staff assess and treat your family member's breathlessness?	69.00	17.90	61.67	18.58	1.38	0.173
4. How well did the ICU staff assess and treat your family member's agitation?	43.50	22.48	38.33	18.58	0.81	0.421
5. How well did the ICU staff show an interest in your needs?	45.00	25.75	38.33	20.85	0.92	0.364
6. How well did the ICU staff provide emotional support?	42.50	22.73	41.67	18.09	0.13	0.897
7. How well did the ICU staff co-ordinate the care of your family member?	73.00	18.10	75.00	18.90	-0.37	0.711
8. How well did the ICU staff treat you with courtesy, respect, and compassion?	69.50	21.00	58.33	15.43	1.91	0.061
9. How well do you think the nurses cared for your family member?	74.00	18.87	70.00	14.02	0.76	0.451
10. How often did the nurses communicate to you about the family member's condition?	50.50	23.95	48.33	24.03	0.31	0.760
11. How well do you think the doctors cared for your family member?	79.00	17.76	81.67	17.59	-0.51	0.611
12. The atmosphere in the ICU was?	35.00	22.59	40.00	18.42	-0.78	0.437
13. The atmosphere in the waiting room was?	24.50	19.88	20.00	16.90	0.79	0.430
14. How satisfied were you with the level or amount of health care your family member received in the ICU?	79.00	15.45	78.33	16.00	0.15	0.885
15. How often did the doctors talk to you about your family member’s condition?	54.00	22.79	55.00	25.35	-0.15	0.885
16. How willing were staff to answer your questions?	44.00	26.03	33.33	24.40	1.41	0.163
17. How capable were the intensive care staff of giving you explanations that you understood?	66.00	20.05	63.33	16.00	0.47	0.639
18. How honest do you think the information you received about your family member’s condition was?	62.50	19.07	75.00	21.13	-2.17	0.034
19. How well did ICU staff inform about what was happening to your family member and why things were done?	68.00	21.45	71.67	22.89	-0.57	0.569
20. How consistent was the information you received about your family member’s condition?	51.50	29.63	51.67	19.97	-0.02	0.984
21. Did you feel included in the decision-making process?	29.50	33.38	35.00	36.35	-0.55	0.585
22. Did you feel supported during the decision-making process?	40.00	30.72	48.33	32.00	-0.91	0.365
23. Did you feel that you had control over the care of your family member?	42.00	31.72	36.67	37.64	0.55	0.586
24. Did you have adequate time to concerns addressed and questions answered	87.50	12.63	96.67	8.80	-2.62	0.011

The mean family satisfaction CARE (FS-CARE) score was higher compared to family satisfaction decision-making (FS-DM) for male gender, overall age, previous h/o ICU admission, living in the same city as the hospital, contact with the patient, and survival status as shown in Table 5.

**Table 5:** Demographic factors in relation to family satisfaction FS-CARE, family satisfaction CARE; FS-DM, family satisfaction decision-making

		FS-CARE		FS-DM	
		Mean	±SD	Mean	±SD
Gender	Male	59.07	4.82	54.50	7.87
	Female	56.31	4.15	56.67	8.27
Age	20-30 years	58.86	4.33	56.34	8.91
	31-40 years	59.25	4.57	52.73	7.54
	41-50 years	57.14	5.38	54.84	7.72
	51-60 years	60.12	6.25	52.50	6.52
	>60 years	55.81	3.68	56.25	5.20
	Overall	58.43	4.79	55.00	7.95
Previous h/o ICU admission	Yes	58.10	3.90	50.96	8.51
	No	58.52	5.02	56.01	7.56
Lives in the same city as the hospital	Yes	57.30	5.14	50.45	11.61
	No	58.66	4.74	55.93	6.77
Contact with patient	Lives with patient	58.55	4.38	55.35	7.95
	Weekly	57.15	6.44	60.00	5.00
	Monthly	59.58	6.25	51.14	6.65
	Less than one year	57.14	0.00	52.50	7.07
	yearly	56.55	5.38	57.92	10.42
Survival status	Yes	58.13	5.01	55.07	7.06
	No	58.86	4.53	54.91	9.21

## Discussion

The main aim of this study was to collect the views of patients admitted to the ICU. The selection of these patients was based on their clinical characteristics, and they were asked to complete a questionnaire in which they were asked to rate their experience during their stay in the facility. The hospital, a government medical college-tertiary center in an urban area, received patients from lower educational and socio-economic strata. Factors such as financial exhaustion and last-resort visits were significant in the patient profile.

Our study found that relatives were similarly satisfied with the patient's treatment and the decision-making process. However, their satisfaction with their need for information was lower. This observation is consistent with previous surveys [8-9]. Carlson et al. attributed this finding to the fears and concerns of family members and the workload of ICU medical staff [17]. According to Carlson et al., healthcare staff may experience anxiety that makes it difficult for them to communicate with patients' family members [17]. Regular family discussions ensure that all family members receive the necessary information. In our institute, regular family discussions are conducted separately by the ICU physicians, the primary admitting physicians, and the nursing staff. As a result, the nursing staff in the ICU is not informed of the full aims of the treatment. In addition, there is the possibility of a discrepancy in the information provided to family members. Hwang et al. and Pochard et al. found evidence in their study that inadequate and inconsistent communication by ICU staff was associated with an increased likelihood of anxiety in family members [18,19].

Heyland et al. study consistently found FSs of over 75 in all aspects [20]. A survey in a South Indian private hospital found high patient satisfaction with care, information, and decision-making processes. In this survey, we found that the level of satisfaction with patient care was 78.85, the level of information needed about the patient was 65.38, the level of satisfaction with the decision-making process was 30.77, and the level of overall satisfaction with patient care was 57.00. The lowest score of 23.46 was for the question "atmosphere in the waiting room," followed by 30.77 for the question "involved in the decision-making process." The question "atmosphere in the intensive care unit" received a score of 36.15, while the question "control over your family member's care" received a score of 40.77. The question "Staff answered your questions" received a score of 41.54 and the question "Support in decision making" received a score of 41.92. The questions "ICU staff assessed and responded to your family member's agitation" and "ICU staff supported you emotionally" both received a score of 42.31. The question "ICU staff took an interest in your needs" received a score of 43.46. Medical staff in the ICU are exposed to high levels of stress and overwhelm due to the frequent and rapid admission and discharge of patients. Therefore, their efforts to inform patients' families are not optimal. Many members expressed the feeling of meeting all the requirements of the patients free of cost or having separate ICU pharmacies at affordable prices and having more visiting hours to see the patient.

The study by Frivold et al. found that family members are satisfied with patient treatment and care but less satisfied with themselves and the communication skills of critical care staff. Factors like ICU stay length and family member gender predict this outcome [9]. In our study, the mean FS-CARE score was higher compared to FS-DM for male gender, overall age, previous h/o ICU admission, living in the same city as the hospital, contact with the patient, and survival status.

In the current survey, the majority of family members expressed satisfaction with the patient's care and the competence and expertise of the nurses and doctors. The level of satisfaction with the communication and care provided to family members was lower. Inadequate communication and lack of consistency by critical care staff can increase the likelihood of depression symptoms in family members [18,19]. It is crucial for all ICU team members to be aware of each patient's treatment goals and hold regular family meetings before leaving the ICU to ensure necessary information is provided [21].

The study differs from previous research by selecting polytrauma patients instead of those from medical intensive care units (MICU), cardiology ICU, and neurology ICU. MICU patients are often elderly and chronically ill. Polytrauma patients are often young, healthy individuals, putting a significant burden on family decision-makers, potentially influencing their role in the decision-making process. Stricker et al. research indicates a link between severe illness and higher satisfaction with family care, which is reflected in our study by the fact that patients whose APACHE score (general measure of disease severity) was higher scored a higher satisfaction score [21]. Bray et al. study suggests that a higher ratio of registered nurses to ICU patients positively impacts patient and family outcomes [22]. Our ICU nurse-to-patient ratio varies from 1:2 to 1:4 with a total number of doctors six to eight.

Family members play a significant role in end-of-life patient care in the ICU, with increased promotion and research papers exploring its importance. This involvement has been highlighted in various studies by various researchers [23,24]. Involving all family members in the communication process is crucial, regardless of the patient's condition or outcome [25]. Effective communication helps them understand the patient's condition, including diagnosis, prognosis, and treatment risks and benefits. This understanding is essential for family members to act as surrogate decision-makers [26].

The survey revealed dissatisfaction among participants with the waiting area atmosphere, a trend consistent with previous research studies [23,27]. The study by Bharadwaj et al. found that patients are highly satisfied with their care in the ICU, with a satisfaction score of 59.97 [28]. However, the nurse-patient ratio varies from 1:1 to 1:4, with seven doctors, and nurses facing an excessive workload, resulting in insufficient information being shared with patients' families. 

In our study, mean satisfaction scores for all questions were similar for men and women, with the exception of time taken to respond to questions and concerns, which was significantly higher for women. The psychological well-being of family members affected by ICU stays extends beyond those who have experienced the loss of a loved one. The current approach to supporting deceased patient families is insufficient, with women reporting more aftercare needs than men, and gender is a significant predictor of these needs. Informal caregivers are predominantly women, who receive less support from family and friends than men. Male caregivers are more likely to receive help from those outside their immediate environment. Women are more susceptible to the negative effects of caregiving responsibilities on their health, experiencing higher levels of burden, stress, anxiety, and depression [29]. The study suggests that women should place a greater emphasis on post-ICU monitoring [30]. The study suggests that post-ICU stay aftercare interventions should involve phone calls and ICU visits, with nurses playing a crucial role in providing guidance, promoting physical well-being awareness, and reducing vulnerability, but further research is needed [30]. 

The present study provides new evidence on family members' recommendations for effective aftercare interventions. These recommendations include telephone calls several weeks after the ICU stay to provide an opportunity to clarify information and facilitate revisits to the ICU to help clarify issues. Further research is needed to identify the most appropriate support structure for family members who require follow-up care after their ICU experience.

The study has limitations; the questionnaire was available in Hindi and English language but some of the members were illiterate. The translation of the questionnaire for illiterate may have led to some misinterpretation, which may have affected the results of the study. Although a person is assigned the responsibility of the questionnaire, he/she is not available round the clock, which affects their experience, and thus the response reflected in the questionnaire. Stress, depression, and over-burden might influence their satisfaction with the quality of ICU care and management. A smaller sample size may have resulted in a limited correlation between various parameters and the level of satisfaction. The FS-ICU questionnaire may not comprehensively capture the cultural and family values specific to Indian households, which affects the results of the study.

## Conclusions

Although families were very satisfied with the ICU stay, several areas were identified as having potential for improvement. The present study shows that the quality of treatment and communication during hospitalization is a major factor in the need for follow-up care. This underlines the need for a constant focus on communication skills in the training of nurses and doctors and their practical training in the ICU. Participation in decision-making, especially by family members of survivors, was identified as an area for improvement. This is the first study in India to select polytrauma patients for FS-ICU scoring, emphasizing quality treatment and patient family satisfaction level as key quality indicators. Future research is required to investigate the factors that influence family involvement in decision-making in the ICU including socio-cultural, organizational, and contextual factors. We recommend more research to be conducted in India focusing on family satisfaction with involvement in the decision-making in ICU considering the unique racial, cultural, ethnic, and linguistic differences in India.
